# Plant-derived nanoparticles as alternative therapy against Diarrheal pathogens in the era of antimicrobial resistance: A review

**DOI:** 10.3389/fmicb.2022.1007115

**Published:** 2022-12-15

**Authors:** Tesleem Olatunde Abolarinwa, Daniel Jesuwenu Ajose, Bukola Opeyemi Oluwarinde, Justine Fri, Kotsoana Peter Montso, Omolola Esther Fayemi, Adeyemi Oladapo Aremu, Collins Njie Ateba

**Affiliations:** ^1^Antimicrobial Resistance and Phage Biocontrol Research Group, Department of Microbiology, School of Biological Sciences, Faculty of Natural and Agricultural Sciences, North-West University, Mmabatho, South Africa; ^2^Department of Chemistry, Faculty of Natural and Agricultural Sciences, North-West University, Mmabatho, South Africa; ^3^Indigenous Knowledge Systems Center, Faculty of Natural and Agricultural Sciences, North-West University, Mmabatho, South Africa

**Keywords:** biocontrol, diarrheagenic bacteria, multidrug resistance, nanotechnology, therapeutic agent, antimicrobial agent, metal-free plant-derived nanoparticles

## Abstract

Diarrhea is a condition in which feces is discharged from the bowels frequently and in a liquid form. It is one of the frequent causes of morbidity and mortality in developing countries. The impact of Diarrhea is worsened by the increasing incidence of antimicrobial resistance among the causative agents, and this is now categorized as a global healthcare challenge. Antimicrobial resistance among Diarrheal pathogens also contributes to extended infection durations, and huge economic loss even in countries with advanced public health policies. The ever-increasing incidence of antimicrobial resistance including the contraindications arising from the administration of antibiotics in some Diarrheal cases highlights a crucial need for the development of novel non-antibiotic alternative agents for therapeutic and biocontrol applications. One such intervention includes the application of plant-derived nanoparticles (PDNPs) with novel antimicrobial properties. Given their small size and large surface area to volume ratio, PDNPs can attack target bacterial cell walls to generate reactive oxygen species that may simultaneously disrupt bacteria cell components such as DNA and proteins leading to cell damage or death. This potential can make it very difficult for pathogenic organisms to develop resistance against these antibacterial agents. In this review, we provide a critical overview on the antimicrobial resistance crisis among Diarrheagenic bacteria. We also discuss the evidence from the existing literature to support the potential associated with the use of PDNPs as alternative therapeutic agents for multidrug resistant and antibiotics administer contraindicated bacteria that are associated with Diarrhea.

## Introduction

Diarrhea is among the most frequent cause of morbidities and mortalities in humans even in countries with advanced healthcare facilities. The global increase in antimicrobial resistance among Diarrheal pathogens is a great public health concern requiring urgent attention (Troeger et al., 2018). In 2019, the Global Burden of Disease (GDB) indicated that Diarrhea was the third and fifth leading cause of disability adjusted life years (DALYs) in children below 5 years old and all ages, respectively (Vos et al., 2020; Behera and Mishra, 2022). In South Africa, data from the National Burden of Disease (NBD) indicate that Diarrhea remains among the top 10 diseases associated with high mortalities especially among children (Nannan et al., 2019). The most frequent causative agents of Diarrhea are bacteria (Feuerstein et al., 2022), viruses (rotavirus, norovirus, astrovirus, and adenovirus), and protozoa (*Giardia*, *Entamoeba histolytica*, and *Cryptosporidium* species; Behera and Mishra, 2022). The transmission of Diarrheal pathogens is often through the consumption of contaminated food or water (Jung et al., 2020). The clinical signs and symptoms of Diarrhea include severe abdominal pain, vomiting, watery stool, bloody stool, and fever (Wei et al., 2020).

In some patients, Diarrhea may be self-limiting and can be treated by antibiotics to avoid life-threatening complications (Taylor et al., 2017). However, the administration of antibiotics is contraindicated for Diarrhea caused by some bacteria strains including those belonging to the *E. coli* serotypes O157 (Yoh et al., 1997; Wong et al., 2000; Mullish and Williams, 2018; Endtz, 2020a; Hwang et al., 2021). This limits the use of antibiotics in treating Diarrhea. In addition, there is a rapid emergence of multiple drug resistance (MDR), excessive drug resistance (XDR), and pan drug resistance (PDR) among Diarrheal pathogens (Huchin et al., 2017; Nkansa-Gyamfi et al., 2019; Ammar et al., 2021). This resistance is mainly attributed to the misuse of antimicrobial agents among humans and animals (Aminshahidi et al., 2017). The rapid increase of antimicrobial resistance among Diarrheal causative organisms is a global healthcare concern accountable for an increase in morbidity and mortality rates, extended duration of infections in susceptible individuals, and huge financial loss (IHME, 2020).

The significant increase in antimicrobial resistance coupled with the contraindications associated with the administration of antibiotics especially in Diarrheal cases highlights a crucial need for the development of novel strategies aimed at not only combating infections but also controlling the development and spread of antibiotic resistance. This review focuses on antimicrobial resistance among Diarrheagenic bacteria and highlights the public health crises it presents to both human and animal medicine. The potential antibacterial properties of PDNPs as alternative therapeutic agents for multidrug resistant bacteria and with specific focus on Diarrheal pathogens are also highlighted.

## The causative agents of Diarrhea

**Figure 1:**
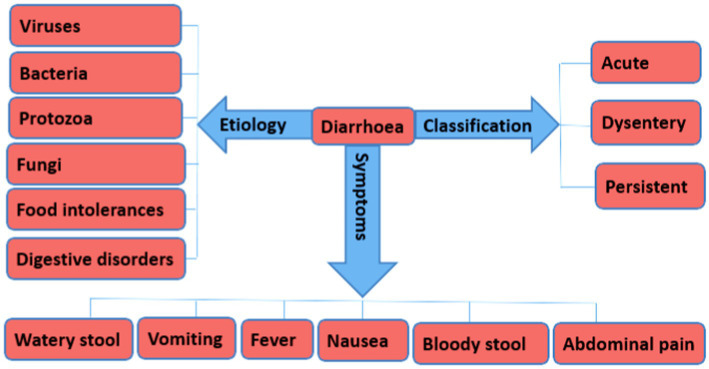
An overview of the different forms of Diarrhea, etiology, and symptoms.

Generally, viruses, bacteria, and protozoa are the frequent causative agents of Diarrhea among humans and animals (Figure 1). Other factors include food allergies and intolerances, long-term use of medications, and digestive disorders, such as ulcerative colitis, microscopic colitis, and small intestinal bacterial overgrowth (Troeger et al., 2018; Zhou et al., 2021). However, Diarrhea caused by bacterial pathogens is known to cause high mortality. In addition, the focus of this review on bacteria pathogens as is mainly due to the rapid increase in the development of antimicrobial resistance among bacterial species thus limiting treatment options.

### Bacteria

Bacteria remain the leading cause of Diarrhea and significantly contribute to approximately 31% of all Diarrheal pathogen’s cases (IHME, 2020). Particularly, *Escherichia coli*, *Vibrio cholerae, Shigella, Salmonella, Campylobacter*, and *Clostridium* species (Table 1) are most frequently associated with cases of Diarrhea (Kim et al., 2015). In 2019, different bacteria pathogens were associated with global morbidities and mortalities resulting from Diarrheal complications (IHME, 2020; Murray et al., 2020).

**Table 1:** Global illness and death due to Diarrheagenic bacteria complications in 2019 (IHME, 2020; Murray et al., 2020).

Diarrheagenic bacteria	Pathogenic species	No. of morbidity	No. of mortality
*Campylobacter* species	*Campylobacter jejuni* and *Campylobacter coli*	7,307,839	139,079
*Clostridium* species	*Clostridium difficile* and *Clostridium perfringens*	870,814	32,134
*Escherichia coli*	Enteroaggregative *E*. *coli*, Enteroinvasive *E*. *coli*, Enterotoxigenic *E*. *coli*, Enteropathogenic *E*. *coli*, Shiga-toxin producing *E*. *coli*, and Diffusely adherent *E*. *coli*	3,374,777	60,414
*Salmonella* species	*Salmonella typhi, Salmonella paratyphi, Salmonella enteritidis,* and *Salmonella typhimurium*	4,269,216	61,646
*Shigella* species	*Shigella dysenteriea*, *Shigella flexneri*, *Shigella sonni*, and *Shigella boydii*	10,602,910	148,202
*Vibrio* species	*Vibrio cholerae*	7,134,552	117,240

#### Escherichia coli

*Escherichia coli* forms part of the normal intestinal microbiota of humans (Gomes et al., 2016; Vidal et al., 2019). However, some *E*. *coli* strains have acquired virulence genes that allow them to cause moderate to severe diseases depending on host-pathogen interactions (Gomes et al., 2016). To date, several pathogenic *E*. *coli* strains are known to be significant causative agents of Diarrhea (Gomes et al., 2016; Vidal et al., 2019) and are collectively termed Diarrheagenic *E*. *coli* (DEC; Table 1). These DEC are grouped into different pathological types (pathotypes) based on differences in the virulence gene they harbor, namely diffusely adherent *E*. *coli* (DAEC), enterotoxigenic *E*. *coli* (ETEC), enteropathogenic *E*. *coli* (EPEC), enteroinvasive *E. coli* (EIEC), enteroaggregative *E*. *coli* (EAEC), and enterohemorrhagic *E*. *coli* (EHEC; Zhou et al., 2018). Enteropathogenic *E*. *coli* remains one of the most frequent causes of Diarrhea in children under the age of 5 in developing countries (Hu et al., 2015). The ability of EPEC to cause Diarrhea is associated with the potential to produce attaching and effacing lesions (Hu et al., 2015). The possession of aggregative adherence (*aag*) gene determinants by EAEC, invasive plasmid antigen H (*ipaH*) gene by EIEC and heat-stable (*st*) and heat-labile (*lt*) genes by ETEC account for the pathogenicity of these strains as well as their ability to cause Diarrhea (Mare et al., 2021). Enterohemorrhagic *E*. *coli* or STEC can cause acute inflammation of the intestine that results to bloody Diarrhea. This group of pathogenic *E*. *coli* are capable of producing Shiga toxins with two principal types, stx_1_ and stx_2_ currently known and characterized (Zhang J. et al., 2020). In addition, other putative variants of stx_2_ such stx_2a_, stx_2c_, and stx_2d_ have been reported and their association with disease in human have been fully documented (Baranzoni et al., 2016).

#### *Shigella* species

Shigellosis is a common cause of Diarrhea, accounting for about 148,202 deaths globally (Vos et al., 2020). *Shigella* species are the second leading cause of mortality among children in low and middle-income countries, accounting for approximately 60,000 deaths per year (Rogawski McQuade et al., 2020). *Shigella* species have low infectious dose and thus they are recognized as pathogens of severe public health concern when compared to other enteric pathogens. Malnutrition and poor health care facilities exacerbate the impact of infections caused by *Shigella* species especially in developing countries (Sheikh et al., 2019). The transmission of these pathogens usually occur through the consumption of contaminated food and/or water (Abebe et al., 2020). *Shigella* species that are frequently associated with Diarrhea include *S*. *boydii, S*. *dysenteriae, S*. *sonnei,* and *S*. *flexneri* (Aminshahidi et al., 2017; Abbasi et al., 2019). The pathogenicity of *Shigella* species is also associated with their ability to produce two distinct toxins, namely, Shigella enterotoxin 1 and Shigella enterotoxin 2, which are structurally and genetically similar to the Shiga toxins produced by STECs (Yaghoubi et al., 2017). The enterotoxin 2 produced by *S*. *dysenteriae* facilitates invasion of epithelial cells of the small intestines, while the enterotoxin 1 accounts for the watery phase of Diarrhea (Yaghoubi et al., 2017).

#### *Campylobacte*r species

Globally, *Campylobacter* species are some of the most common pathogens that cause foodborne diseases including Diarrhea (Li Y. et al., 2018). The most frequently isolated *Campylobacter* species from Diarrhea patients are *C*. *jejuni* and *C*. *coli*, accounting for about 500 million gastrointestinal illnesses every year (Zhang P. et al., 2020). Infections caused by *Campylobacter* species are generally mild but can be severe in younger children, the elderly, or immunocompromised individuals thus requiring urgent attention to avoid complications, such as bacteremia, irritable bowel syndrome, hepatitis, and pancreatitis (Same and Tamma, 2018). The complications, duration, and high incidence of Diarrhea caused by *Campylobacter* species make it socio-economically significant problem requiring urgent attention (Endtz, 2020b).

#### *Salmonella* species

In 2019, Diarrhea resulting from *Salmonella* species was estimated to be 4.2 million cases with 61,646 deaths (Vos et al., 2020). Particularly in Europe, the most common *Salmonella* species that causes Diarrhea is *S*. *enteritidis*, where it accounts for eight out of every 10 cases of Diarrhea caused by *Salmonella* species (Wei et al., 2019; Yue et al., 2020). Other commonly isolated salmonellae include *S*. *typhimurium*, *S*. *Paratyphi*, and *S*. *Typhi* (Terefe et al., 2020). The clinical symptoms that are most common in Diarrheal patients infected by *Salmonella* species include fever, vomiting, nausea, abdominal pains, and in some cases bloody stool (Wei et al., 2020). The most vulnerable group comprises pregnant women, children below 5 years, and elderly people as well as immunocompromised individuals (Demb et al., 2014).

#### *Vibrio* species

*Vibrio* species remain an important and a prevalent causative agent of Diarrheal cases that contributes to morbidity in humans of all ages globally (Ganesan et al., 2020). *Vibrio cholerae* is the species that is most often isolated from patients with Diarrhea and it has been responsible for a number of outbreaks worldwide making it a pathogen of significant public health concern (Bashar and Soundappan, 2022). Its socio-economic impact indicates the need for highly effect control strategies that aim to contribute to efforts made by different organizations such as WASH to curb the problem worldwide (Igere and Ekundayo, 2020). *Vibrio cholerae* causes an acute Diarrheal illness by invading the intestine of humans and it accounts for about 2,900,000 cases with 95,000 deaths annually (Ganesan et al., 2020). Diarrhea caused by *V*. *cholerae* is usually mild without symptoms but may progress to life-threatening complications if left untreated. With a relatively short incubation period of between 24 and 48 h before the onset of disease, cholera presents approximately a 25–50% mortality rate in humans, thus the need to prevent cross-contamination and the occurrence of disease (Igere and Ekundayo, 2020). However, cholera is often predictable and preventable. The disease can ultimately be eliminated especially during outbreaks by ensuring access to clean water and sanitation facilities, as well as good hygiene practices provided the entire population.

## Diarrhea therapy and antibiotics resistance crisis

Although Diarrheal illness may be self-limiting and can be manage by oral rehydration, antimicrobial treatment is recommended for seriously-ill patients and immunocompromised individuals to avoid complications (Taylor et al., 2017). Unfortunately, a large proportion of individuals suffering from Diarrhea rarely report cases to hospitals but rather resorts to self-medication (Ferdous, 2018). This practice has been highly discouraged especially if the causative agent is *E*. *coli* strain belongs to the STEC serotype O157:H7 (Hwang et al., 2021; Ramstad et al., 2021; Tarr and Freedman, 2022). Antibiotic therapy is contraindicated in the treatment and management of Diarrhea infections caused by STEC because there is evidence that antibiotics may increase the risk of developing more life threatening complications, such as haemolytic uremic syndrome (HUS), haemorrhagic colitis, and thrombotic thrombocytopenic purpura (TTP) that have all been associated with renal failure (Hwang et al., 2021; Ramstad et al., 2021; Tarr and Freedman, 2022). This supports the view to ensure that appropriate tests are conducted to correctly determine the identities of causative agents before antibiotic therapy. In addition, the global misuse of antibiotics among individuals has led to a significant increase of antibiotic-resistant pathogens against most of the available potent antibiotics rendering them ineffective (Aminshahidi et al., 2017). Also, some of these pathogens are resistant to carbapenem antibiotics, which are considered to be a “last resort” drug for the management of infections caused by multiple antimicrobial resistant pathogens (Livermore et al., 2020). Furthermore, some of the Diarrheagenic bacteria have displayed multiple drug resistance (MDR) while others have acquired extensive drug resistance (XDR) determinants (Table 2). The Diarrhea cause by antibiotic-resistant pathogens can lead to severe illnesses, extended hospital stays, increase in healthcare costs, socio-economic burden as well as treatment failures (Rahim et al., 2021). The death rate in humans resulting from complications caused by resistant Diarrheagenic bacteria has increased globally (Vos et al., 2020; Mac Kinnon et al., 2021). In 2019, the global death caused by notable Diarrheagenic bacteria such as *E*. *coli*, *V*. *cholerae, Shigella, Salmonella, Campylobacter*, and *Clostridium* species were 60,414, 117,240, 148,202, 61,646, 139,079, and 32,134, respectively (IHME, 2020). This rapid increase in antimicrobial resistance couple with the contraindications associated with the administration of antibiotics in Diarrheal cases highlight a crucial need for the development of novel and alternative therapeutic antibacterial agents. This will contribute to the global action plan on antimicrobial resistance developed by WHO requiring each country to establish its own antimicrobial resistance strategic framework to curb the spread of antimicrobial resistance (Mendelson and Matsoso, 2015). Against this background, several intervention strategies have been proposed to mitigate the spread of antibiotic resistance. One such intervention includes the application of medicinal plant derivatives nanoparticles. The application of nanoparticles as alternatives for drug resistant bacteria contributes to the aim of reducing the incidence of infections and an investment in countering antimicrobial resistance.

**Table 2:** Diarrheagenic bacteria classification based on their antimicrobial resistance capacity.

Diarrheagenic bacteria	Antimicrobial category	No. of isolate tested	MDR	XDR	PDR	References
*Campylobacter* species	AMI, CAR, CEP, FLU, GLY, LIN, MAC, PEN, SUL, and TET	120	90	25	5	Ammar et al. (2021)
		235	28	69	3	El-Aziz et al. (2020)
		322	105	211	6	El-Naenaeey et al. (2021)
*Clostridium* species	AMI, CAR, CEP, FLU, GLY, LIN, MAC, PEN, SUL, and TET	30	21	8	1	Gharieb et al. (2021)
*Escherichia coli*	AMI, CAR, CEP, FLU, GLY, LIN, MAC, PEN, SUL, and TET	8,713	7,336	1,368	8	Feuerstein et al. (2022)
		14,336	3,870	3,440	716.8	Nkansa-Gyamfi et al. (2019)
*Salmonella* species	AMI, CAR, CEP, FLU, GLY, LIN, MAC, PEN, SUL, and TET	271	133	81	40	Inbaraj et al. (2022)
*Shigella* species	AMI, CAR, CEP, FLU, GLY, LIN, MAC, PEN, SUL, and TET	12	2	8	2	Huchin et al. (2017)
*Vibrio cholera*	AMI, CAR, CEP, FLU, GLY, LIN, MAC, PEN, SUL, and TET	31	6	2	0	Lee et al. (2018)

MDR; Multi-drug resistance, XDR; Extensive drug resistance, PDR; Pan-drug resistance, AMI; Aminoglycosides, CAR; Carbapenems, CEP; Cephalosporins, FLU; Fluoroquinolones, GLY; Glycopeptides, LIN; Lincomycins, MAC; Macrolides, PEN; Penicillins, SUL; Sulfonamides, TET; Tetracyclines.

## Alternative therapeutic approaches and strategies for multidrug resistant Diarrheal pathogens

The significant and rapid increase in antimicrobial resistance among bacteria pathogens including those associated with Diarrhea pose a serious concern, especially to public health globally. This advocates for the need to develop new alternative therapeutic approaches to curb the spread of antimicrobial resistance (Harbarth et al., 2015). Recently, a number of antibacterial agents and strategies have been used to control pathogenic antibiotic-resistant bacteria (Aldayel, 2019). These attempts utilize either chemical (agents and compounds) or natural (microbes and plant extracts) methods. To date, there is evidence of the advantages that natural agents from microbes and plants possess over chemical agents, and this explains why natural agents are considered as novel alternative therapeutic approaches for combating antimicrobial resistance even among Diarrheal pathogens (Hobernik and Bros, 2018; Aldayel, 2019; Kalsoom et al., 2020). In fight against antimicrobial resistance, vaccine, bacteriophages, and nanoparticles provide some hope (Aldayel, 2019). However, more research is still needed to assess the safety and suitability of these agents. Moreover, the development of sustainable and highly effective antimicrobial agents will not only contribute to the global action plan on antimicrobial resistance, but also provide opportunities to generate data on the mechanisms of action of these natural agents.

### Nanotechnology and nanoparticles as a novel approach in combating antibiotics resistant Diarrheal pathogens

**Figure 2:**
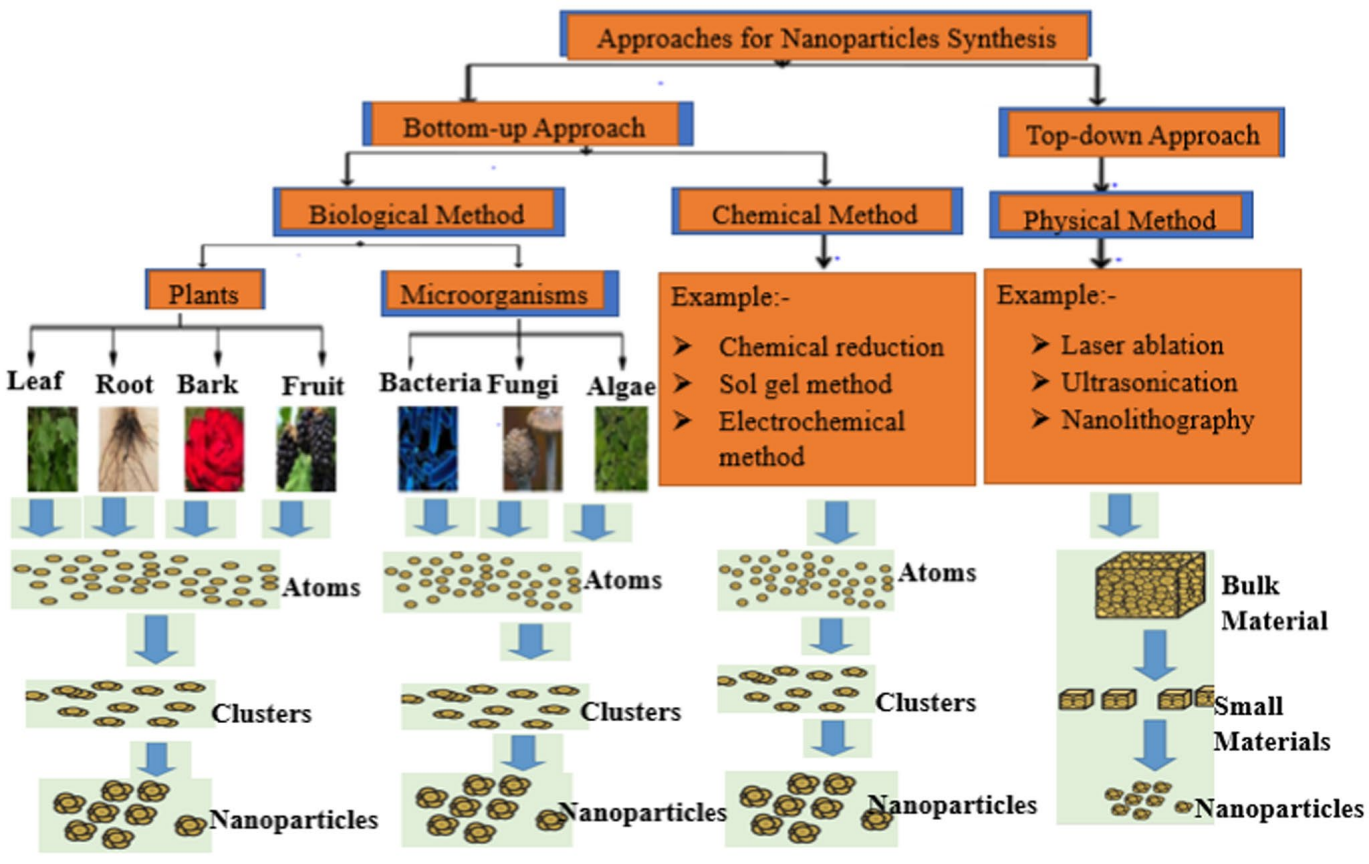
Overview of the methods used for nanoparticles production.

Nanoparticles (NPs) are small particles that range between 1 and 100 nm in size and possess a large surface area to volume ratio. The large surface to volume ratio enhances their physicochemical properties compared to macro range particles. The small size to the large surface area to volume ratio of NPs influences their binding and reactive abilities, making them suitable antimicrobial and sensory agents. These attributes facilitate their use in biolabeling, filters, microelectronics, and catalysis (Kaabipour and Hemmati SJBJoN., 2021). Generally, NPs are produced using chemical, physical, and biological methods (Figure 2). Physical synthetic methods for NPs involve thermal decomposition, laser irradiation, and electrolysis that usually require very high temperatures, are energy-intensive and also require high-cost vacuum systems with other supporting equipments (Khan et al., 2022). Chemical methods typically involve the use of chemicals, which may be harmful to humans under certain conditions (Karimi-Maleh et al., 2021). Due to these limitations, recent studies typically focus on biological synthetic methods due to their non-toxicity, cheap, and environmentally friendly nature (Zhang et al., 2016; Nemidkanam and Chaichanawongsaroj, 2022). Plants are a major source of different types of phytochemicals with numerous biomedical applications and the extracts are used to treat various diseases. Different parts of plants including leaves, fruits, seeds, stems, flowers, roots, barks, and fruit peels have been used in the synthesis of various types of nanoparticles (Venkat Kumar and Rajeshkumar, 2018). Plant-based NP green synthesis is now regarded as a gold standard approach among the green biological techniques owing to its ease of use and the diversity of plants (Hano and Abbasi, 2022). The most popular PDNPs synthesis techniques involves the reduction of metals, such as copper, gold, silver, nickel, platinum, zinc, and titanium with the plant extracts serving as reducing, capping, and stabilizing agents (Ahmad et al., 2020). Findings from several studies have reported high efficacy of various metal-based PDNPs against a variety of multidrug resistant pathogenic bacteria (Banerjee et al., 2022). However, the development of various side effects including argyrosis, argyria, liver damage, kidney obstruction, and eye irritation, in patients limits the usage of metal-based PDNPs for oral treatment of drug resistant bacteria in human medicine (Korani et al., 2015). Metal-free NPs can be produced from plant extracts by using the differential ultracentrifugation technique (Iravani and Varma, 2020; Kim et al., 2021). The production of metal-free PDNPs has recently gained significant attention because plants contain materials such as terpenoids, alkaloids, polyphenols, phenolics, sugars, and proteins as metabolites that can be broken down to produce nanoparticles with significant antimicrobial properties. Metal-free PDNPs are environmental friendly, toxic chemical free, cheap, and energy serving (Kharissova et al., 2019; Surendran et al., 2021). The potential to derive metal-free NPs with medicinal properties from plants has gained more attention worldwide (Zhang et al., 2016; Saravanan et al., 2021; Surendran et al., 2021; Liu et al., 2022; Nemidkanam and Chaichanawongsaroj, 2022). Liu et al. (2022) reported the therapeutic ability of metal-free PDNPs on gut microbiota while Xiao et al. (2018) highlighted the therapeutic efficacy of metal-free NPs synthesized from 11 edible fruits and vegetables. Saravanan et al. (2021) reported the antibacterial efficacy of metal-free NPs derived from *Curcuma longa* plant against *E. coli, S. aurous, K. pneumoniae,* and *S. epidermidis*. These attributes highlight and amplify the usefulness and effectiveness of metal-free PDNPs.

#### Metal-free plant-derived nanoparticles as alternative therapy for the treatment of Diarrhea

The production of PDNPs involves the use of plant extracts for synthesizing these unique small-size particles that also have large surface area to volume ratios. Nanoparticles have the potential to react with bacterial cell walls and generate reactive oxygen species (ROS) that can simultaneously disrupt important bacterial cell components such as DNA and proteins, thus leading to cell death (Saravanan et al., 2021). Metal-free PDNPs have been reported to be safe for oral therapy in humans because they are derived from the plants whose components, such as carbohydrates, proteins, and lipids are also vital for survival (Xiao et al., 2018; Großkopf and Simm, 2020; Saravanan et al., 2021). The presence of these compounds makes it easy for metal-free PDNPs to be metabolized and cleared from the intestine either through absorption or defecation (Li H. et al., 2018; Großkopf and Simm, 2020). Several researchers have reported the antimicrobial efficacy of metal-free NPs synthesized from different plants against various pathogenic bacteria (Table 3). A study on NPs synthesized from *Curcuma longa* extracts confirmed its significant antimicrobial efficacy against multidrug-resistant bacteria (Saravanan et al., 2021). Metal-free NPs synthesized from *Lawsonia inermis* extracts had high antimicrobial activities against antimicrobial-resistant *E*. *coli* and *Salmonella* species, which are the most frequently isolated Diarrheal pathogens (Shahshahanipour et al., 2019). Similar reports on the antimicrobial efficacy of PDNPs synthesized from extracts of *Citrus medica* against MDR and biofilm-producing bacteria have also been documented (Selvaraju et al., 2022). The compounds in plants when present in the PDNPs, contribute in the treatment of diseases (Ajose et al., 2022), thus a valuable source for alternative non-antibiotic therapeutic agents. These attributes provide evidence of the therapeutic and biocontrol properties of PDNPs against multidrug resistant bacteria, including those associated with Diarrhea.

**Table 3:** Examples of metal-free plant derived nanoparticles.

Plant	Type	Size (nm)	Component	Target	References
*Ananas comosus*	C-NPs	2.4	C, O, N, S	*Escherichia coli, Bacillus cereus, Staphylococcus aureus, Pseudomonas aeruginosa*	Surendran et al. (2021)
*Brassica oleracea*	C-NPs	32.4	C, O, N	Gastrointestinal tract	Deng et al. (2017)
*Camellia sinensis*	C-NPs	5	C. O. N	*Escherichia coli, Staphylococcus aureus*	Ma et al. (2020)
*Cannabis sativa*	C-NPs	5	C	*Escherichia coli, Staphylococcus aureus*	Raina et al. (2020)
*Capsicum annuum*	C-NPs	3.1	C, O	Tumor cell	Vasimalai et al. (2018)
*Cinnamomum verum*	C-NPs	3.4	C, O	Tumor cell	Vasimalai et al. (2018)
*Citrus medica*	C-NPs	4.5	C, O, N	*Pseudomonas aeruginosa*	Selvaraju et al. (2022)
*Curcuma longa*	C-NPs	2.6	C, O, N	*Escherichia coli, Klebsiella pneumonia, Staphylococcus aureus, Staphylococcus epidermidis*	Saravanan et al. (2021)
*Impatiens balsamina*	C-NPs	3.25	C, CL, N	*Escherichia coli, Bacillus cereus, Staphylococcus aureus, Pseudomonas aeruginosa, Salmonella*	Liu et al. (2021)
*Lawsonia inermis*	C-NPs	5	C, O	*Escherichia coli, Staphylococcus aureus*	Shahshahanipour et al. (2019)
*Osmanthus fragrans*	C-NPs	6.5	C, N, O	*Escherichia coli, Staphylococcus aureus*	Ma et al. (2020)
*Trapa bispinosa*	C-NPs	7.5	ND	Bacteria Pathogens	Mewada et al. (2013)

C-NPs, Carbon nanoparticles; H, hydrogen; C, carbon; O, oxygen; N, nitrogen; and ND, not detected.

#### Factors influencing the antimicrobial activity of nanoparticles

**Figure 3:**
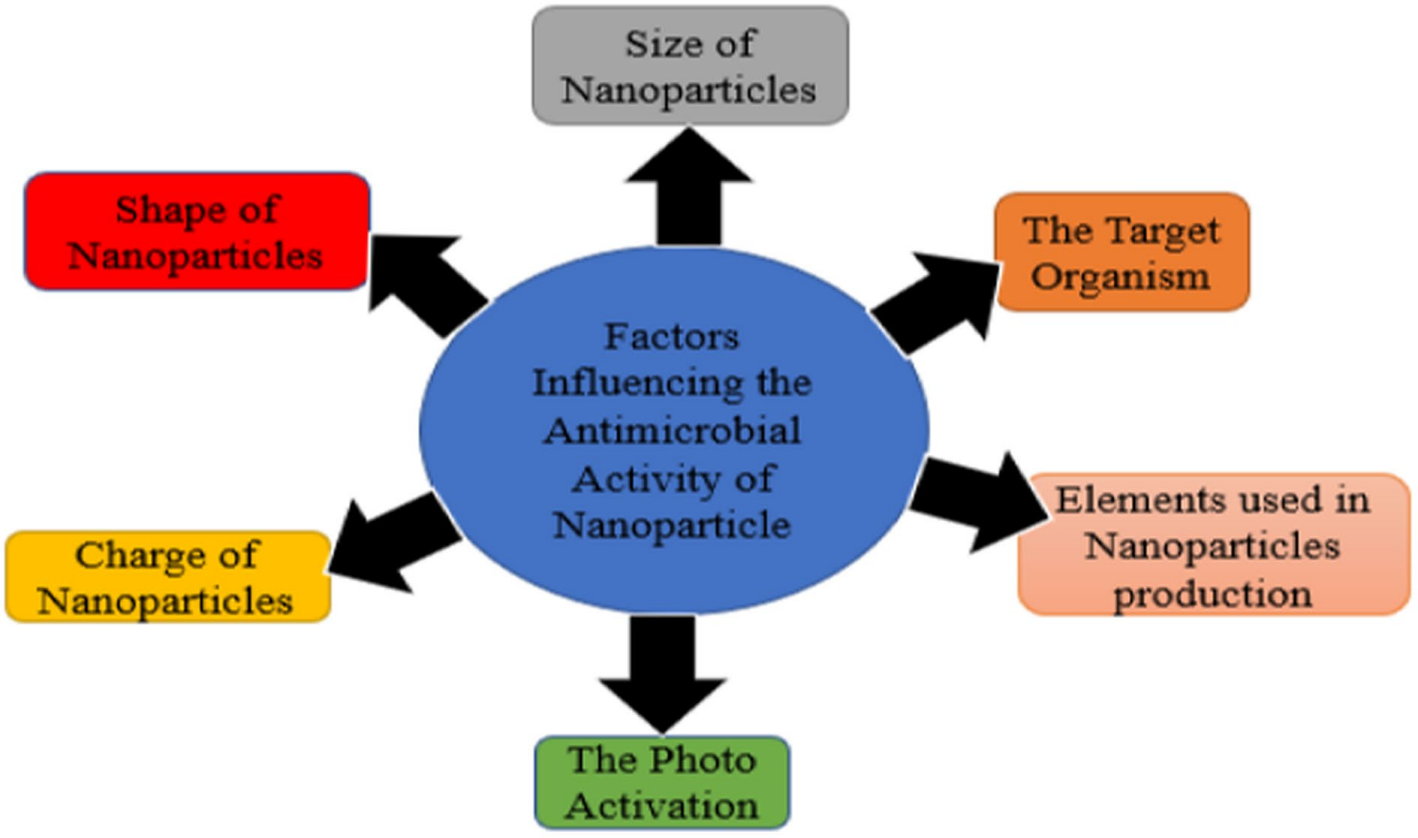
Factors influencing antimicrobial activity of nanoparticles.

Antimicrobial activities of metal-free NPs can be influenced by various factors which include, size, elements used in the production, shape of nanoparticle, charge of NPs (Sun et al., 2021), and target microorganisms (Figure 3).

##### Size of nanoparticles

The small size and large surface-to-volume ratio of smaller metal-free PDNPs enhance their physical and chemical properties when compared with larger NPs (Saravanan et al., 2021). The smaller the size, the larger the surface-area-to-volume ratio; hence, the greater the antimicrobial activity. The high antimicrobial activities of smaller PDNPs results to larger surface area to volume ratios thus improving contact with the cell wall of microorganisms (Saravanan et al., 2021). Contact with cell wall and the production of ROS results in the destruction of essential cell biomolecules, such as DNA, proteins, and lipids leading to cell death. These attributes give smaller NPs an edge over their larger counterparts in inhibiting the growth of microorganisms (Sun et al., 2021). Shahshahanipour et al. (2019) reported that small size (5 nm) of NPs derived from *Lawsonia inermis* plant caused damage of *E. coli* and *S. aureus* cell wall.

##### Shape of nanoparticles

Different shapes for PDNPs have been reported by several researchers (Saravanan et al., 2021; Sun et al., 2021). These include spherical, sheets, plates, tubes, cubes, rods, and triangles, but the most common shape is spherical. Analysis has revealed that NPs that has more exposed surface is less stable and require less energy to form oxygen vacancies, thereby has higher antimicrobial activity. Several researchers have demonstrated that NPs with cube or rod shape are more effective than other shapes, due to their exposed surface and oxidation level.

##### Charge of nanoparticles

The charge of NPs usually plays important role in antimicrobial activity. Studies had revealed that positively charge metal-free PDNPs, can disrupt electron transport chain function (Sun et al., 2021). Electron transport chain contains ubiquinone, which is important for aerobic respiration. Analysis on *E. coli* with single deletion had confirmed that mutated bacteria on ubiquinone biosynthesis related genes were more susceptible to the positively charge NPs. The exposure of the bacteria to NPs results to ROS generation that inhibits bacteria growth (Saravanan et al., 2021). In addition, positively charge NPs is more attracted electrostatically to negative charge bacterial cell wall, making it to be more antimicrobial effective than negatively charge metal-free PDNPs. Notwithstanding, some negative charge PDNPs have been reported to have more antibacterial efficacy (Manzano and Vallet-Regí, 2020).

##### Target organisms

Generally, bacteria are classified into Gram-positive and Gram-negative base on their cell wall structure. Peptidoglycan is the most important component of bacterial cell wall, which serves as protection against external force, and it is thicker in Gram-positive bacteria than Gram-negative (Rohde, 2019). Nanoparticles have been revealed to have higher antimicrobial activities against Gram-negative bacteria than Gram-positive (Sun et al., 2021). It had been confirmed that thickness in peptidoglycan that makeup cell wall is the reason this phenomenon exists. The cell wall of Gram-positive bacteria, such as *Staphylococcus aureus* composed of peptidoglycan which is 80 nm thick (Pasquina-Lemonche et al., 2020). This thickness may delay the damage of Gram-positive cell wall by PDNPs compare with cell wall of Gram-negative bacteria such as *E*. *coli* that possess peptidoglycan which is 8 nm thick (Pasquina-Lemonche et al., 2020). In addition, Gram-negative bacteria cell wall is coated with negatively charged lipopolysaccharide which has higher affinity for positive charge ion discharge by most of the metal-free PDNPs (Sun et al., 2021). This higher affinity for positive charged ion by Gram-negative bacteria cell wall results into increase in ion uptake and build-up that damage the cell wall of Gram-negative bacteria faster than Gram-positive bacteria due to absent of lipopolysaccharide (Sun et al., 2021). Wang et al. (2020) reported that NPs derived from *Artemisia argyi* have antimicrobial activities against only Gram-negative bacteria (*E. coli* and *P. aeruginosa*) but not Gram-positive bacteria (*S. aureus* and *Bacillus subtilis*). However, some PDNPs that have higher antimicrobial activities against Gram-positive bacteria than Gram-negative had been reported (Shahshahanipour et al., 2019). Liu et al. (2021) confirmed that positive PDNPs have more antimicrobial properties against Gram-positive bacteria (*S. aureus* and *B. subtilis*) than Gram-negative bacteria (*E. coli* and *Salmonella*).

#### Antibacterial activity of plant-derived nanoparticles

**Figure 4:**
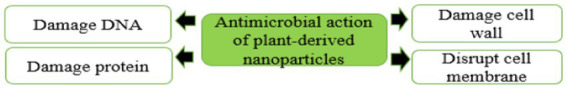
Antimicrobial action exhibited by plant-derived nanoparticles.

Owing to their small size, the effectiveness of PDNPs as an antimicrobial agent has resulted in an increasing number of studies exploring the efficacy of their antimicrobial mechanisms. The small size and large surface area to volume ratio gives PDNPs the potential to damage bacteria cell wall (Shahshahanipour et al., 2019; Wang et al., 2020) and generates ROS (Saravanan et al., 2021), which disrupt DNA, protein, and cell membrane functions (Figure 4). The ability of PDNPs to alter the metabolic activity of the bacteria signifies an enormous advantage in eradicating them (de Barros et al., 2021). The antimicrobial effectiveness of PDNPs is accomplished when they contact microbial cells *via* a receptor ligand, Van der Waals forces, hydrophobic interactions, or electrostatic attraction (de Barros et al., 2021). The accumulation of PDNPs across the microbial cell and its metabolic pathways influences its structure and function. It uses ROS mechanisms to interfere with the bacteria’s cell components, such as DNA, protein, and cell membrane. This interaction may lead to cell death due to increased osmotic pressure (de Barros et al., 2021; Saravanan et al., 2021). PDNPs exhibit various modes of action on target bacterial cells (Table 4), including a disruption of cell membrane function, protein inhibition, DNA synthesis, and damage to the cell wall.

**Table 4:** Multiple antimicrobial action of plant-derived nanoparticles.

Plant	Type	Charge	Size (nm)	Target	Active part	Mechanisms of action	Reference
*Curcuma longa*	C-NPs	-	2.6	Cell wall	Small size	Cell wall damage	Saravanan et al. (2021)
				Protein	ROS generation	Protein disruption	
				DNA	ROS generation	DNA disruption	
*Lawsonia inermis*	C-NPs	-	5	Cell wall	Small size	Cell wall damage	Shahshahanipour et al. (2019)
				Protein	ROS generation	Protein disruption	
				DNA	ROS generation	DNA disruption	
*Ananas comosus*	C-NPs	-	2.4	Cell wall	Small size	Cell wall damage	Surendran et al. (2021)
				Protein	ROS generation	Protein disruption	
				DNA	ROS generation	DNA disruption	
Impatiens balsamina	C-NPs	+	3.25	Cell wall	Small size	Cell wall damage	Liu et al. (2021)
				Protein		Protein disruption	
				DNA		DNA disruption	
					ROS generation		
					ROS generation		

ROS, reactive oxygen species; C-NPs, carbon nanoparticles; −, negative charge; +, positive charge.

##### Disruption of bacteria cell wall

**Figure 5:**
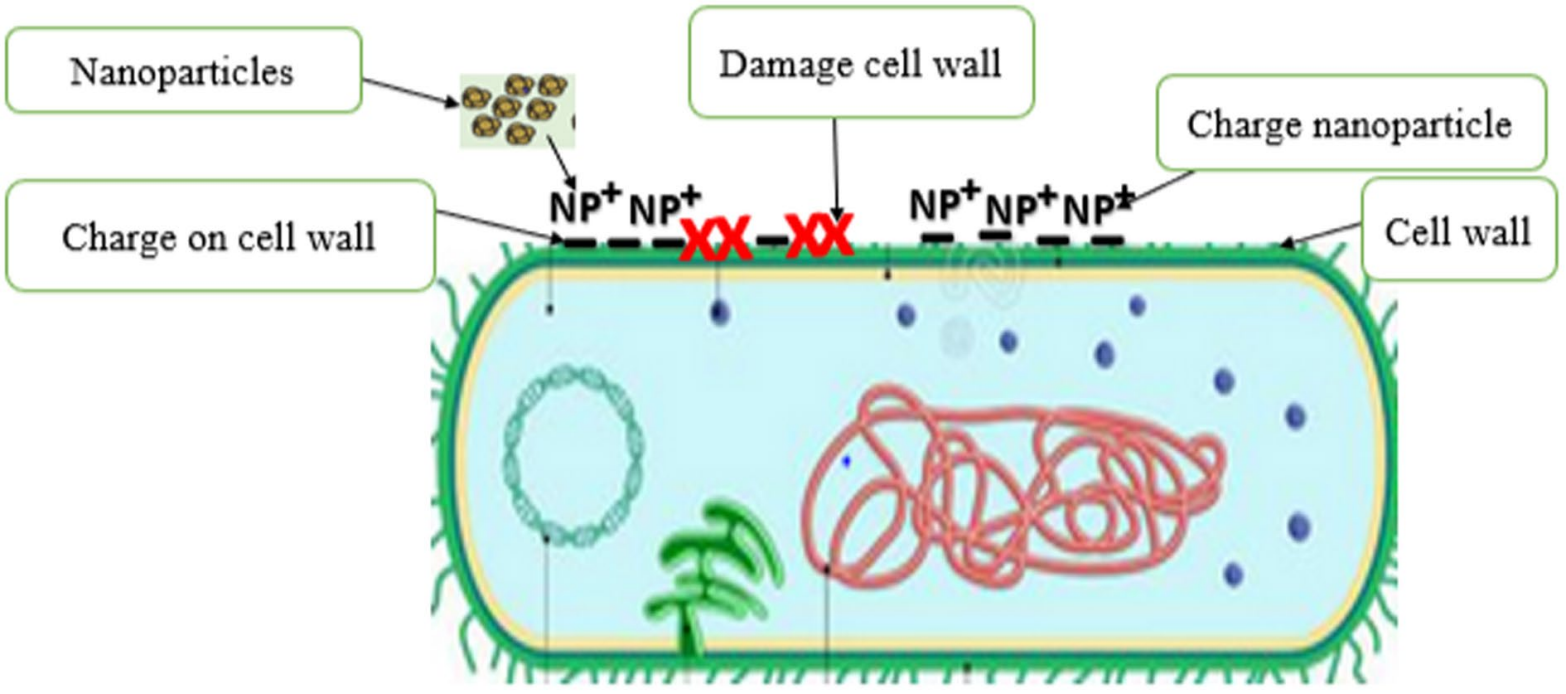
Disruption of bacteria cell wall by plant derived nanoparticles.

The first and most important defense mechanism that microorganisms typically use as a mode of resistance to antimicrobial agents is their cell walls and membranes. The cell wall plays a vital role in keeping the cell viable by protecting its organelles. The cell wall comprises lipopolysaccharides (LPSs), lipoproteins, peptidoglycan, and phospholipids. The wall forms a defensive barrier and thus prevents harmful substances from the external environment from entering (Auer and Weibel, 2017). However, microorganisms have a charge on the surface of their cell walls, which can attract PDNPs when applied as an antimicrobial agent (Shahshahanipour et al., 2019; Saravanan et al., 2021). The charged PDNPs generally bind with the charged functional cell wall groups, such as carboxyl and phosphate groups, in a process called biosorption, resulting in cell walls being structurally weakened and lyses, leading to the microorganism’s cell death (Figure 5). Existing evident indicate that PDNPs have better efficacy against Gram-negative bacteria relative to Gram-positive bacteria (Wang et al., 2020). Ma et al. (2020) reported that the accumulation of *Osmanthus fragrans* (PDNPs) on bacterial cell wall led to the cell wall destruction.

##### The DNA and protein damage

**Figure 6:**
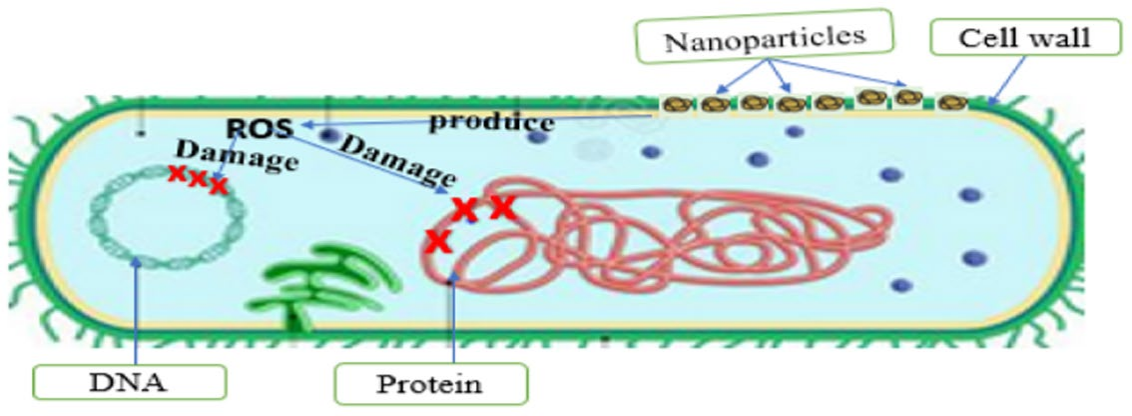
DNA and protein damage.

The interaction of PDNPs with microbial cells may lead to cell death due to DNA denaturation, synthesis inhibition, active cell enzyme inhibition, protein synthesis blockage, and gene expression alteration through the generation of ROS (Surendran et al., 2021). Saravanan et al. (2021) confirmed that NPs derived from *Curcuma longa* used ROS as main antimicrobial mode of action against *Escherichia coli, Klebsiella pneumonia, Staphylococcus aureus,* and *Staphylococcus epidermidis.* When NPs react with bacterial cell wall, ROS are slowly released and absorbed through the cell membrane (Shahshahanipour et al., 2019). ROS-induced oxidative stress is an important antimicrobial mechanism of NPs that has powerful positive redox potential (Shahshahanipour et al., 2019; Saravanan et al., 2021). Different types of NPs produce different types of ROS by reducing oxygen molecules. There are four types of ROS namely superoxide radical, hydroxyl radical (OH), singlet oxygen (O_2_), and hydrogen peroxide (H_2_O_2_), each with different levels of antimicrobial activity. The main causes of ROS production are restructuring, defect sites, and oxygen vacancy in the crystal, which occur due to the interaction of PDNPs with microbial cells, causing an unbalanced state (Figure 6). This unbalanced state causes oxidative stress, damaging or obstructing the microorganism’s organelles’ functions. Oxidative stress has been identified as a major contributor to alteration in the permeability of the microorganism’s cell membrane, leading to cell membrane malfunction and damage. The NPs interaction with the cell membrane ultimately results in the loss of membrane integrity because of intracellular oxidative stress. Furthermore, an increasing number of studies have shown that ROS plays a key role in the disruption of bacteria DNA and protein, which is a key mechanism in microbial cell death (Shahshahanipour et al., 2019; Saravanan et al., 2021). Saravanan et al. (Saravanan et al., 2021), reported that the ROS produce by metal-free PDNPs (*Curcuma longa*) played a significant role in eradicating the target bacteria by disrupting their DNA and protein.

## Effect of plant-derived nanoparticles on host microbiota

The usage of PDNP as an antimicrobial agent is gaining momentum among researchers, and its numerous benefits in various fields, including medicine, have been established (Akintelu and Folorunso, 2020; Manzano and Vallet-Regí, 2020). Although a lot of *in vitro* work has been done by researchers to prove the efficacy of PDNP as an antimicrobial agent, its application as an alternative therapy in the field of medicine has not been well established for safety purposes (Su and Kang, 2020). The assessment of PDNP is essential for a proper therapeutic result and effective clinical use to avoid complications or the potential for negative interactions with host microbiota (Vakili-Ghartavol et al., 2020). Microbiota interacts intimately with their hosts, influencing immune responses, food absorption, and energy metabolism (Hacquard et al., 2015; Brugman et al., 2018). Healthy hosts with beneficial microbiota harbor diverse mutualistic and commensal microbes. The ability of PDNP to disrupt host-associated microbiota raises serious concerns regarding its application as an alternative medicine (Missaoui et al., 2018). As a result, pharmacokinetic and toxicological investigations on the influence of PDNP on the host microbiota are required before its use. Understanding the link between the physicochemical and structural features of PDNPs and host microbiota reactivity and interaction is very important (Missaoui et al., 2018; Brinkmann et al., 2020). This information will aid in the intelligent design of PDNPs while closing the knowledge gap about their host microbiota toxicity (Missaoui et al., 2018).

## Conclusion and future perspectives

This study provides a detailed overview on the antimicrobial resistance crisis among Diarrheal pathogens. Globally, there is a rapid emergence of a multidrug, extensive drug, and pan-drug resistance among Diarrheal pathogens. The calls to develop new, active, environmentally friendly alternative therapies remain pertinent. Research indicates that PDNAs lie at the frontal of possible alternative treatments. A thorough overview of recent developments in the derivation of NPs from various plant parts has been summarized. The merits of PDNPs over physical and chemical techniques of producing NPs, such as being free of harmful chemicals, devoid of metals, environmentally benign, affordable, and energy-serving, were also highlighted. This review also discussed the antibacterial mode of action of PDNPs. The PDNPs sizeable surface-area-to-volume ratio and highly charged surfaces enable them to interact with bacteria cell wall and generate ROS that simultaneously disrupt the bacterial cell membrane, proteins, and DNA. This concurrent antimicrobial action of PDNPs can make it difficult for bacteria to develop resistance. This evidence demonstrates that PDNPs could be developed and used as alternative therapeutic agents against multidrug resistant bacteria, including those associated with Diarrhea.

Despite the numerous benefits of PDNPs in biomedicine, the knowledge about their safety to human health remains limited. Up to date, there is little information about the cytotoxicity of PDNPs. In addition, several *in vitro* assessments of antibacterial efficacy of PDNPs have been done with little or no *in vivo* information. Thus, more efforts are needed for stringent investigation on the cytotoxicity and *in vivo* application of PDNPs to confirm their safety for therapeutic use.

## Author contributions

CA, AA, and OF: conceptualization, resources, supervision, and editing. TA, DA, BO, JF, KM, and CA: writing–review and editing. TA: writing–original draft preparation. All authors contributed to the article and approved the submitted version.

## Funding

We are grateful to the North-West University and the Department of Microbiology, School of Biological Sciences for the financial support.

## Conflict of interest

The authors declare that the research was conducted in the absence of any commercial or financial relationships that could be construed as a potential conflict of interest.

## Publisher’s note

All claims expressed in this article are solely those of the authors and do not necessarily represent those of their affiliated organizations, or those of the publisher, the editors and the reviewers. Any product that may be evaluated in this article, or claim that may be made by its manufacturer, is not guaranteed or endorsed by the publisher.
